# Half-day trading and spillovers

**DOI:** 10.1186/s11782-021-00097-7

**Published:** 2021-02-17

**Authors:** Yifan Chen, Limin Yu, Jianhua Gang

**Affiliations:** 1 School of Economics and Management, Tsinghua University, Beijing, 100084 China; 2 National School of Development, Peking University, Beijing, 100871 China; 3 School of Finance, Renmin University of China, Beijing, 100872 China; 4 China Financial Policy Research Center, School of Finance, Renmin University of China, Beijing, 100872 China

**Keywords:** Spillover effects, Semi-day transaction, Volatility, Multivariate GARCH model, Stock market

## Abstract

This paper investigates the linkage of returns and volatilities between the United States and Chinese stock markets from January 2010 to March 2020. We use the dynamic conditional correlation (DCC) and asymmetric Baba–Engle–Kraft–Kroner (BEKK) GARCH models to calculate the time-varying correlations of these two markets and examine the return and volatility spillover effects between these two markets. The empirical results show that there are only unidirectional return spillovers from the U.S. stock market to the Chinese stock market. The U.S. stock market has a consistently positive spillover to China’s next day’s morning trading, but its impact on China’s next day’s afternoon trading appears to be insignificant. This finding implies that information in the U.S. stock market impacts the performance of the Chinese stock market differently in distinct semi-day trading. Moreover, with respect to the volatility, there are significant bidirectional spillover effects between these two markets.

## Introduction

Over the past several decades, fast development of international trade has deepened economic connections between countries. Because of the observed economic globalization, the flow of capital between international stock markets has become increasingly active, contributing to the integration of stock markets in different countries. Previous research indicates that financial integration can significantly impact the cost of capital (Bekaert and Harvey 2000; Henry 2000). Understanding the linkage between different financial markets is extremely important for both regulatory agencies and financial institutions.

In this paper, we investigate the linkage of both returns and volatilities between the U.S. and Chinese stock markets. As an emerging market, China has developed rapidly in recent decades. It has become the largest developing country and the second-largest economy in the world. According to the World Federation of Exchanges, as of June 2019, the Shanghai Stock Exchange, Shenzhen Stock Exchange, and Hong Kong Stock Exchange are among the world’s top 10 largest exchanges by market value. Moreover, in 2019, China A-shares were added to FTSE Russell’s global indices and MSCI’s global benchmark equity index. The increasing global influence of the Chinese stock market makes it necessary to determine its correlation with international stock markets. This study will help investors to deepen their understanding of risk management in the Chinese stock market. It will also give out policy implications for governments that are seeking to maintain financial stability and coordinate global regulatory policies.

Specifically, we apply the bivariate dynamic conditional correlation (DCC) and asymmetric Baba–Engle–Kraft–Kroner (BEKK) GARCH models to measure the linkage between the Chinese and U.S. markets. The mean functions of both models, which are the vector autoregression (VAR), are used to test the return spillover effects between the two markets. Moreover, we adopt the bivariate GARCH(1, 1)-DCC and asymmetric GARCH(1, 1)-BEKK specifications as variance functions to exam the volatility spillovers.

To preview our results, there are only unidirectional return spillover effects from the U.S. stock market to China’s stock market from January 1, 2010, to March 31, 2020. Moreover, the U.S. stock market’s spillover effects work differently in China’s next day’s morning trading versus the afternoon trading. The U.S. stock market exerts a consistently positive spillover to China’s next day’s morning trading. The significance of this positive spillover is demonstrated in both the DCC-GARCH and BEKK-GARCH models consistently. However, the U.S. stock market’s impact on China’s afternoon trading appears to be inconsistent. For the volatility spillovers, there are bidirectional spillover effects between these two markets. Furthermore, we complement the core analysis with a case study of the impact of the COVID-19 pandemic using daily stock transactions from January 1, 2020, to March 31, 2020. First, we find results that are consistent with the above-stated main conclusions of this paper. In other words, during this period, the U.S. stock market still had a positive spillover to China’s next day’s morning trading. Second, and most importantly, the Chinese stock market exerted significant return spillovers back onto the U.S. stock market during that period.

This study contributes to the existing literature in two areas. First, we provide up-to-date empirical evidence on the spillover effects between the Chinese and U.S. stock markets using the most recent data from January 1, 2010, to March 31, 2020. We also conduct a case study of COVID-19, which is the most recent global pandemic event. Because the COVID-19 pandemic occurred so suddenly and so recently, how it has impacted the stock markets is not fully covered in the existing empirical studies. By conducting this case study, we aim to fill this gap to some extent by providing the most recent empirical evidence of the impact of COVID-19 on the capital markets, especially on the interactions between stock markets across countries. Our analysis builds on the work of Wang and Firth (2004), which focuses on the spillovers from developed international markets to the Chinese stock market. However, in contrast, our analysis focuses on a more recent period when the Chinese market became more influential in the international markets compared with earlier times. Compared to their results, we find more significant and greater spillover coefficients between the U.S. and Chinese stock markets, which provides new evidence of international financial market integration. Apart from finding the deepening impact from the U.S. to the Chinese stock market, we also detect that the influence of the Chinese stock market on the U.S. stock market has been becoming stronger and stronger in recent years. These findings echo recent policies designed to open the Chinese financial market wider to the remainder of the world and make it easier for foreign investors to invest. The Shanghai-Hong Kong Stock Connect and Shenzhen-Hong Kong Stock Connect were also launched in recent years, increasing the degree of participation of international capital. Thus, we investigate the reason for the stronger spillovers between the two markets. We find that the launches of Shanghai-Hong Kong Stock Connect and Shenzhen-Hong Kong Stock Connect as well as the Northbound Capital of foreign investment into Chinese domestic equity market have a significantly positive impact on the stronger spillover effects between the Chinese and U.S. stock markets over time. Our finding has potentially important implications for policy makers to coordinate global integration regulatory policies. As the spillover effects are strengthened between the U.S. and Chinese stock markets, crisis and volatility will also become more contagious between different markets. As in the case study of COVID-19, the shock that hit the Chinese stock market spilled over significantly back to the U.S. stock market. In the background of macro-prudential regulation, our results highlight the importance of strengthening the coordination of the policies of different countries and maintaining financial stability internationally.

Second, our study provides additional insights into the information transmission mechanism of the Chinese stock market. All of the previous studies (Hill et al. 1990; Li and Zhang 2008; Tse et al. 1996; Wang and Firth 2004) have focused only on the whole trading day spillovers between markets, and little attention has been paid to the intraday information transmission mechanisms between the U.S. and Chinese stock markets. In contrast, we divide the trading day of the Chinese stock market into the morning and afternoon transaction segments. In this way, we can differentiate information transmission in different transaction periods within a day and capture the unique characteristics of the Chinese stock market. We find that the U.S. stock market has a significantly positive impact on the Chinese stock market’s next day’s morning trading. However, the U.S. stock market’s spillover is inconsistent on Chinese stock market’s next day’s afternoon trading.^1^ To our knowledge, our paper is the first to differentiate the spillover effects in the different trading segments in China. In contrast to previous studies that found general spillovers from the U.S. to Chinese stock market, our paper provides more specific evidence that the spillover effects work in the morning trading but not the afternoon trading. This finding allows us to better understand the information contained in the Chinese stock market.

The remainder of this paper is organized as follows. Section 2 describes the methods used to analyze the return and volatility spillover effects. Section 3 provides the statistical analysis. Section 4 shows the empirical results. We conclude the paper in Section 5.

## Methodology

### Linear model

Following Chow et al. (2011), we construct a simple linear model to test the impact of the U.S. stock market on the Chinese stock market:

1 $$ {R}_{m,t}^c={\beta}_{m,0}+{\beta}_{m,1}{R}_{m,t-1}^c+{\beta}_{m,2}{R}_{t-1}^{s\&p}+{\varepsilon}_{m,t}, $$

2 $$ {R}_{a,t}^c={\beta}_{a,0}+{\beta}_{a,1}{R}_{a,t-1}^c+{\beta}_{a,2}{R}_{t-1}^{s\&p}+{\varepsilon}_{a,t}, $$

3 $$ {R}_{d,t}^c={\beta}_{d,0}+{\beta}_{d,1}{R}_{d,t-1}^c+{\beta}_{d,2}{R}_{t-1}^{s\&p}+{\varepsilon}_{d,t}, $$

where ${R}_{m,t}^c$, ${R}_{a,t}^c$, and ${R}_{d,t}^c$ denote the semi-day logarithmic return in the morning, semi-day logarithmic return in the afternoon and daily logarithmic return of the CSI 300 Index (CSI300), respectively, and ${R}_t^{s\&p}$ represents the daily return of the S&P 500 Index (SPX). *β*_*i*, 2_ measures the return spillovers from the U.S. stock market to the Chinese stock market. The null hypothesis is that the performance of the U.S. stock market on day *t*–1 (${R}_{t-1}^{s\&p}$) cannot predict the return of the Chinese stock market on day *t*; in other words, for all *i = m*, *a*, *d*, *β*_*i*, 2_ = 0.

This linear model assumes that *ε*_*i*, *t*_ is a white noise process, neglecting heteroskedasticity. We construct more specific models based on different assumptions in Section 2.2.

### The multivariate GARCH model

To better measure the comovements between these two markets, we build a multivariate GARCH (MGARCH) model with DCC and asymmetric BEKK specifications in conditional variance functions. Before illustrating the conditional variance function, we use the function mentioned in Section 2.1 and build our conditional mean function, as follows:

4 $$ {R}_{i,t}={\mu}_i+{\Gamma}_i{R}_{i,t-1}+{\varepsilon}_{i,t},\kern0.4em i=m,a,d, $$

where ${R}_{i,t}={\left({R}_{i,t}^c,{R}_t^{s\&p}\right)}^{\prime }$, ${R}_{i,t}^c$ and ${R}_t^{s\&p}$ are defined as above. In addition, we have

$$ {\mu}_i=\left(\begin{array}{c}{\mu}_{i,1}\\ {}{\mu}_{i,2}\end{array}\right),{\Gamma}_i=\left(\begin{array}{cc}{\gamma}_{i,11}& {\gamma}_{i,12}\\ {}{\gamma}_{i,21}& {\gamma}_{i,22}\end{array}\right),\kern0.5em {\varepsilon}_{i,t}=\left(\begin{array}{c}{\varepsilon}_{i,1t}\\ {}{\varepsilon}_{i,2t}\end{array}\right), $$

where *μ*_*i*_ is the constant vector, and *Γ*_*i*_ is the matrix of parameters that represent return spillover effects between the two markets. To be more specific, *γ*_*i*, *mn*_, the diagonal element of *Γ*_*i*_, measures the impact of its past returns, while *γ*_*i*, *mn*_, the off-diagonal element of *Γ*_*i*_, captures the influence of the past return of market *n* on the current return of market *m*. Thus, we should pay close attention to the significance of *γ*_*i*, *mn*_. The variable *ε*_*i*, *t*_, whose conditional covariance matrix is *H*_*i*, *t*_, is the random error on day *t*. *H*_*i*, *t*_ is described by the DCC-GARCH and asymmetric BEKK-GARCH models.

Bollerslev et al. (1988) propose an MGARCH model, known as the General Vech GARCH model:

$$ vech\left({H}_t\right)= vech(C)+\sum \limits_{i=1}^q{A}_i vech\left({\varepsilon}_{t-i}{\varepsilon_{t-i}}^{\prime}\right)+\sum \limits_{i=1}^q{G}_i vech\left({H}_{t-i}\right), $$

where *vech* is the operator that stacks the lower triangular portion of a symmetric matrix into a vector.

However, the number of parameters to be estimated in this MGARCH is typically large. In addition, some restrictions are imposed on parameters to satisfy the positive definite property of the conditional variance matrix.

To solve these problems, many parametric formulations are introduced for the structure of the conditional variance-covariance matrices. Tse and Tsui (2002) propose the DCC-GARCH model, and Engle and Kroner (1995) introduce the BEKK model, which have been widely used, and both of these models effectively solve these problems.

#### DCC-GARCH model

Based on Tse and Tsui (2002), the DCC-GARCH model is applied for the conditional variance function. The mean function is

$$ {R}_{i,t}={\mu}_i+{\Gamma}_i{R}_{i,t-1}+{\varepsilon}_{i,t},\kern0.4em i=m,a,d, $$

and the variance function is

5 $$ {\displaystyle \begin{array}{c}{\upvarepsilon}_{i,t}\mid {\Omega}_{t-1}\sim D\left(0,{H}_{i,t}\right)\\ {}{H}_{i,t}={D}_{i,t}^{\prime }{R}_{i,t}{D}_{i,t}\end{array}}, $$

where $$ {H}_{i,t}=\left(\begin{array}{cc}{h}_{i,11,t}& {h}_{i,12,t}\\ {}{h}_{i,21,t}& {h}_{i,22,t}\end{array}\right),{D}_{i,t}=\left(\begin{array}{cc}{h}_{i,11,t}& 0\\ {}0& {h}_{i,22,t}\end{array}\right),{R}_{i,t}=\left(\begin{array}{cc}1& {\rho}_{i,12,t}\\ {}{\rho}_{i,12,t}& 1\end{array}\right) $$. *H*_*i*, *t*_, *D*_*i*, *t*_ and *R*_*i*, *t*_ are the conditional covariance, variance, and correlation matrix of *ε*_*i*, *t*_, respectively, and *Ω*_*t* − 1_ denotes the conditional information set at time *t* − 1.

The conditional variance of each market in *D*_*i*, *t*_ follows the univariate GARCH(1, 1) process. In other words, we have

6 $$ {h}_{i, jj,t}={c}_j+{\alpha}_j{\varepsilon}_{i,j,t-1}+{\beta}_j{h}_{i, jj,t-1},\kern1em j=1,2, $$

and *R*_*i*, *t*_ depends on

7 $$ {R}_{i,t}=\left(1-{\theta}_1-{\theta}_2\right){R}_i+{\theta}_1{\Psi}_{i,t-1}+{\theta}_2{R}_{i,t-1}, $$

where *R*_*i*_ is a symmetric positive definite constant matrix, and *Ψ*_*i*, *t*_ is a matrix of *ε*_*i*, *t*_ whose elements represent weighted averages of residuals (see the concrete elements of *Ψ*_*i*, *t*_ in Tse and Tsui (2002)). This structure of *H*_*i*, *t*_ guarantees that *R*_*i*, *t*_ is positive definite.

The above DCC-GARCH model is estimated with a two-step method. Applying the DCC-GARCH model, we can calculate the time-varying conditional correlation between the returns of two markets, giving us an excellent opportunity to explore the characteristics of the comovements of the two markets over different periods of time (morning, afternoon and whole day). This approach allows us to not only test the significance of the return spillovers but also assess the strength of the return spillovers. Specifically, the higher the time-varying correlation is, the stronger the linkage between two markets’ returns, and vice visa.

However, since the conditional variance of each market in period *t* is determined by only its variance and error term in period *t*-1, there is no straightforward parameter that can be interpreted as the volatility spillover effects of the two markets. Therefore, we need to build a model to measure the volatility spillovers.

#### Asymmetric BEKK-GARCH model

Since the DCC-GARCH model cannot test the volatility spillover effects, we use the full (unrestricted) BEKK model for the asymmetric responses of the volatility to calculate the *p*-value of the parameters associated with relations in terms of volatility across markets. Our mean function is proposed as follows:

$$ {R}_{i,t}={\mu}_i+{\Gamma}_i{R}_{i,t-1}+{\varepsilon}_{i,t},\kern0.4em i=m,a,d. $$

The variance function is

8 $$ {\displaystyle \begin{array}{c}{\upvarepsilon}_{i,t}\mid {\Omega}_{t-1}\sim D\left(0,{H}_{i,t}\right)\\ {}{H}_{i,t}={C_i}^{\prime }{C}_i+{A_i}^{\prime }{\varepsilon}_{i,t-1}{\varepsilon_{i,t-1}}^{\prime }{A}_i+{B_i}^{\prime }{H}_{i,t-1}{B}_i+{D_i}^{\prime }{\xi}_{i,t-1}^{\prime }{\xi}_{i,t-1}{D}_i\end{array}}, $$

where $$ {C}_i=\left(\begin{array}{cc}{c}_{i,11}& {c}_{i,12}\\ {}0& {c}_{i,22}\end{array}\right),{A}_i=\left(\begin{array}{cc}{a}_{i,11}& {a}_{i,12}\\ {}{a}_{i,21}& {a}_{i,22}\end{array}\right),{B}_i=\left(\begin{array}{cc}{b}_{i,11}& {b}_{i,12}\\ {}{b}_{i,21}& {b}_{i,22}\end{array}\right),{D}_i=\left(\begin{array}{cc}{d}_{i,11}& {d}_{i,12}\\ {}{d}_{i,21}& {d}_{i,22}\end{array}\right) $$.Here, *ξ*_*i*, *t* − 1_ is defined as *ε*_*i*, *t* − 1_ if *ε*_*i*, *t* − 1_ is negative, and 0 otherwise, which shows the impact of negative shocks on the conditional volatility. One advantage of BEKK is that it provides methods for measuring the volatility spillover effects between two markets. According to Eq. (8), the conditional variance of each market is determined by lagged error terms, lagged conditional variance, and lagged shocks from bad news from the two markets. The diagonal parameters in matrices *A*_*i*_, *B*_*i*_ and *D*_*i*_ measure the effects of each market’s past shocks, volatilities, and negative shocks on its current conditional variance, while the off-diagonal parameters in matrices *A*_*i*_, *B*_*i*_ and *D*_*i*_ (*a*_*i*, *mn*_, *b*_*i*, *mn*_ and *d*_*i*, *mn*_) measure the impacts of past shocks, volatilities, and negative shocks of market *m* on the current conditional variance of market *n*. Therefore, if *a*_*i*, 21_ = *b*_*i*, 21_ = *d*_*i*, 21_ = 0, there is no volatility spillover from the U.S. stock market to the Chinese stock market. Similarly, if *a*_*i*, 12_ = *b*_*i*, 12_ = *d*_*i*, 12_ = 0, there is no volatility spillover effect from the Chinese stock market to the U.S. stock market. In addition, another advantage of the BEKK model is that *H*_*i*, *t*_ is positive definite if the diagonal elements of *C*_*i*_ are positive.

## Dataset and variables

We obtain the Chinese and U.S. stock market indices from Wind and Bloomberg, respectively. The indices used are the SPX for the U.S. and the CSI300 for China. Both indices are recorded at the semi-day frequency and are measured in local currencies. The sample period spans from January 1, 2010, to March 31, 2020.

The daily returns of the CSI300 and SPX (${R}_{d,t}^c$ and ${R}_t^{s\&p}$) are computed by the first difference of the logarithm of the closing price of the stock market indices. The semi-day return of the CSI300 in the morning on day *t* (${R}_{m,t}^c$) is obtained by taking the first difference of the logarithm of the closing price at 11:30 GMT + 8 on day *t* and the closing price at 15:00 GMT + 8 on day *t*-1. Similarly, the semi-day return of the CSI300 in the afternoon on day *t* (${R}_{a,t}^c$) is calculated by taking the first difference of the logarithm of the closing price at 15:00 GMT + 8 on day *t* and the closing price at 11:30 GMT + 8 on day *t* +1. To avoid the nonsynchronous trading effects caused by the different festivals of both countries, we select only the data from the days when both markets are traded. The realized volatility of four return series at period *t* is defined as the standard deviation of the returns from day *t*−5 to *t* + 5. All of the variables defined above are shown in Table 1.

**Table 1:** Variable definitions

Variable	Explanations
${R}_{m,t}^c$	Semi-day return of the CSI300 Index in the morning on day* t*
${R}_{a,t}^c$	Semi-day return of the CSI300 Index in the afternoon on day *t*
${R}_{d,t}^c$	Daily return of the CSI300 Index on day *t*
${R}_t^{s\&p}$	Daily return of the S&P 500 Index on day *t*
${Vol}_{m,t}^c$	Realized volatility of the semi-day return of the CSI300 Index in the morning on day *t*
${Vol}_{a,t}^c$	Realized volatility of the semi-day return of the CSI300 Index in the afternoon on day *t*
${Vol}_{d,t}^c$	Realized volatility of the daily return of the CSI300 Index on day *t*
${Vol}_t^{s\&p}$	Realized volatility of the daily return of the S&P 500 Index on day *t*

*Notes.* The returns are all calculated as log returns, i.e., the first difference of the logarithm of the closing prices of the stock market indices. The realized volatility of a return on day *t* is defined as the standard deviation of the returns from day *t *−5 to *t* + 5

Table 2 shows the summary statistics of the return series. The mean of the semi-day CSI300 return in the afternoon transaction segment is the highest. Three return series of the CSI300 have a higher or same standard deviation than that of the SPX. The measures for skewness and kurtosis show that all of the return series are negatively skewed and highly leptokurtic with respect to the normal distribution. The ADF test tells us that all of the return series are stationary processes, which makes the coefficients of the linear model and mean function in the MGARCH models unbiased.

**Table 2:** Summary statistics

Variable	${R}_{m,t}^c$	${R}_{a,t}^c$	${R}_{d,t}^c$	${R}_t^{s\&p}$
Mean (%)	−0.018	0.029	0.012	0.037
SD (%)	1.087	0.910	1.463	1.086
Min (%)	−8.565	−7.292	−8.748	−11.984
Max (%)	5.788	4.835	6.715	9.383
Skewness	−0.524	−0.515	−0.515	−0.680
Kurtosis	8.881	10.369	7.408	20.369
ADF-test	−46.637***	−39.872***	−48.315***	−19.035***

*Notes.* The ADF-test is used to test whether the return series has a unit root. In this table, *, **, and *** denote two-tailed significance at the 10%, 5%, and 1% level, respectively

To preliminarily describe the linkage between the Chinese and U.S. stock markets, we focus on the unconditional correlations of both markets. The correlation matrix is listed in Table 3. The correlation between ${R}_{m,t}^c$ and ${R}_t^{s\&p}$ is higher than that between ${R}_{a,t}^c$ and ${R}_t^{s\&p}$, which indicates that the comovements between the two markets is higher in the morning trading segment and lower in the afternoon.

**Table 3:** Unconditional correlation matrix of returns

Variable	${R}_{m,t}^c$	${R}_{a,t}^c$	${R}_{d,t}^c$	${R}_t^{s\&p}$
${R}_{m,t}^c$	1			
${R}_{a,t}^c$	0.069*** (3.377)	1		
${R}_{d,t}^c$	0.785*** (62.164)	0.672*** (44.481)	1	
${R}_{t-1}^{s\&p}$	0.133*** (6.584)	0.103*** (5.071)	0.133*** (6.584)	1

*Notes.* This table reports the unconditional correlation matrix of semi-day returns of the CSI300 in the morning, semi-day returns of the CSI300 in the afternoon, daily returns of the CSI300 and daily returns of the SPX. The sample period spans from January 1, 2010, to March 31, 2020. The *t*-statistics of the correlations are shown in parenthesis. One, two and three asterisks (*) indicate that the *t*-values are significant at the 0.1, 0.05, and 0.01 level, respectively

**Fig. 1:**
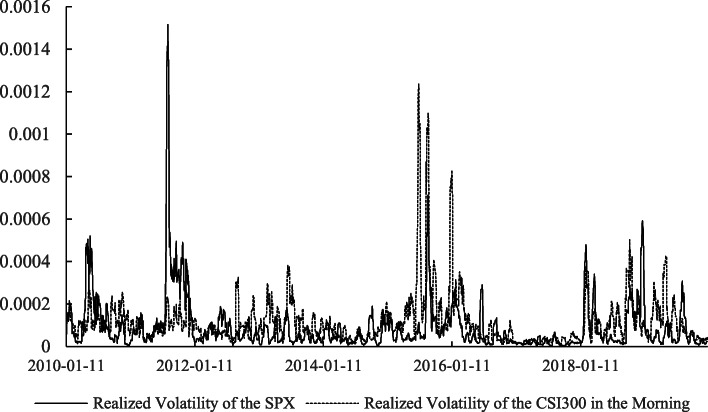
Realized volatility of the SPX and CSI300 in the morning. *Notes.* This figure plots the realized volatilities of CSI300 in the morning and SPX over the whole day. The realized volatility of CSI300 in the morning on day *t* is calculated as the standard deviation of the semi-day morning return of CSI300 from day *t* – 5 to *t* + 5. The realized volatility of SPX on day *t* is defined as the standard deviation of the daily return of SPX from day *t* – 5 to *t* + 5. The data are from the Wind database and span from January 1, 2010, to December 31, 2019 (The realized volatilities from January 1 to March 31, 2020 are much greater than in previous periods. Please refer to Appendix B for details about realized volatilities during that period.)

**Fig. 2:**
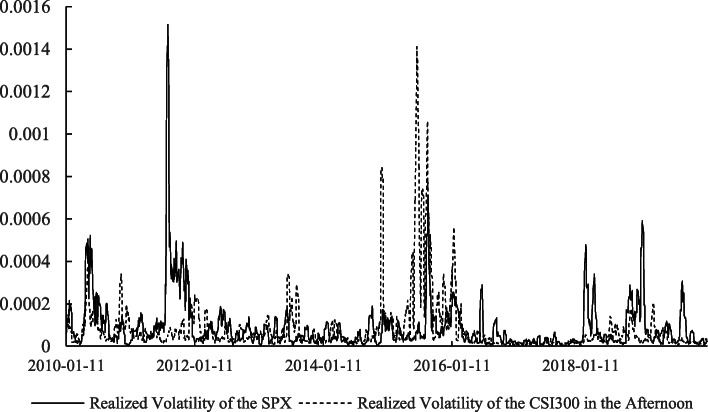
Realized volatility of the SPX and CSI300 in the afternoon. *Notes.* This figure plots the realized volatilities of CSI300 in the afternoon and SPX over the whole day. The data are from the Wind database and span from January 1, 2010, to December 31, 2019

**Fig. 3:**
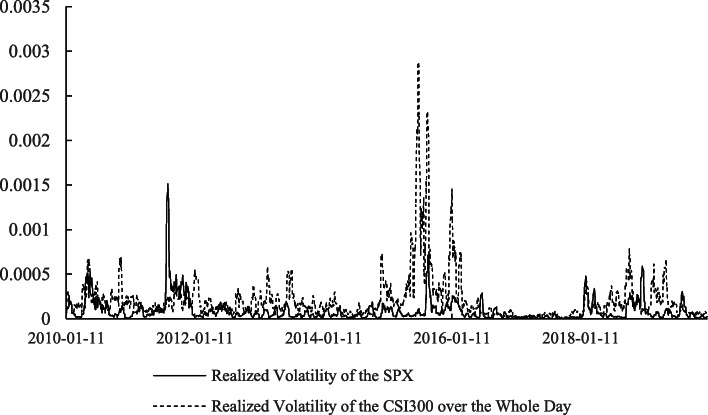
Realized volatility of the SPX and CSI300 over the whole day. *Notes*. This figure plots the realized volatilities of CSI300 and SPX over the whole day. The data are from the Wind database and span from January 1, 2010, to December 31, 2019

Apart from the return, the risk of the stock markets, measured by their volatilities, cannot be omitted. Information about the relations between the realized volatilities of the two markets at different times is extracted, as shown in Table 4. Based on the *t*-statistics in parentheses, we know that all of the correlations are significant at the 1% level. The correlation between the SPX and the CSI300 in the morning trading on the Chinese market is the highest, which indicates that the volatility of the U.S. stock market over the whole day has a closer relationship with the Chinese stock market’s morning trading transactions than its afternoon trading transactions. In addition, comparing the relations between the different realized volatilities of the CSI300 shown in Figs. 1, 2, and 3, we find that the realized volatility of the CSI300 in the morning and the SPX over the whole day tend to move synchronously, which further confirms the inference above.

**Table 4:** Correlation matrix of realized volatilities

Variable	${Vol}_{m,t}^c$	${Vol}_{a,t}^c$	${Vol}_{d,t}^c$	${Vol}_t^{s\&p}$
${Vol}_{m,t}^c$	1			
${Vol}_{a,t}^c$	0.532*** (30.738)	1		
${Vol}_{d,t}^c$	0.843*** (76.740)	0.847*** (78.071)	1	
${Vol}_t^{s\&p}$	0.227*** (11.401)	0.143*** (7.069)	0.148*** (7.318)	1

*Notes.* This table reports the correlation matrix of the realized volatilities of semi-day returns of the CSI300 in the morning (${Vol}_{m,t}^c$), semi-day returns of the CSI300 in the afternoon (${Vol}_{a,t}^c$), daily returns of the CSI300 (${Vol}_{d,t}^c$) and daily returns of the SPX (${Vol}_t^{s\&p}$). The sample period spans from January 1, 2010, to March 31, 2020. The *t-*statistics of the correlations are shown in parenthesis. One, two and three asterisks (*) indicate that the *t*-values are significant at the 0.1, 0.05, and 0.01 level, respectively

## Empirical results

### Linear model

We follow the approach used by Chow et al. (2011). The results are shown in Table 5. Only *β*_*m*, 2_ and *β*_*d*, 2_ are significant at the 1% level, which indicates that the U.S. stock market only exerts a significant influence on the Chinese stock market in the morning but not on the Chinese stock market’s afternoon segment. Analyzing *R*^2^ listed in Table 5, we conclude that the lagged return of the U.S. stock market offers stronger predictability for the returns of the Chinese stock market in the morning than in the afternoon and during whole day. However, the linear model ignores the volatility spillover effects between the two markets. Furthermore, because of significant heteroskedasticity (White-test) in the residuals and the low *R*^2^, we require a more suitable model to test the return and volatility spillover effects between the Chinese and U.S. stock markets.

**Table 5:** Results of the linear model

Dependent variable	Constant	${R}_{i,t-1}^c$	${R}_{t-1}^{s\&p}$	White test	_*R*_^2^
${R}_{m,t}^c$	3.115 × 10^−4^ (2.152 × 10^−4^)	0.017 (0.020)	0.253*** (0.020)	77.440 ***	0.066
${R}_{a,t}^c$	2.934 × 10^−4^ (1.845 × 10^− 4^)	−0.127*** (0.020)	− 0.027 (0.017)	138.170***	0.018
${R}_{d,t}^c$	5.210 × 10^−5^ (2.958 × 10^−4^)	0.010 (0.020)	0.220*** (0.027)	110.310***	0.026

*Notes.* This table reports regressions of the returns of the CSI300 in the morning, afternoon, and whole day on a constant, a one-order-lagged dependent variable (${R}_{i,t-1}^c$) and a one-order-lagged return of the SPX (${R}_{t-1}^{s\&p}$), respectively. The sample period spans from January 1, 2010, to March 31, 2020. The white test is applied to test the heteroskedasticity. Newey-West Robust standard errors are reported in parentheses. One, two and three asterisks (*) indicate that the *t*-values are significant at the 0.1, 0.05, and 0.01 level, respectively

### DCC-GARCH model

To better test the significance and measure the strength of the return spillovers between these two countries’ stock markets, we introduce the DCC-GARCH model. In this model, we use SC to determine the lag order of VAR. Thus, an optimal multivariate VAR(1) is constructed as the conditional mean function. Then, a multivariate GARCH(1, 1) model in the DCC specification is constructed as the conditional variance function. Estimates are summarized in Table 6.

**Table 6:** DCC-GARCH model estimation results

	${R}_{m,t}^c$	${R}_{a,t}^c$	${R}_{d,t}^c$
**Mean equation**			
*γ*_*i*, 11_	0.017 (0.700)	− 0.165*** (− 7.071)	1.063 × 10^− 3^ (0.044)
*γ*_*i*, 12_	0.280*** (10.620)	− 0.044** (− 2.443)	0.238*** (7.130)
*γ*_*i*, 21_	− 0.026 (− 1.103)	9.005 × 10^− 3^ (0.371)	− 0.010 (− 0.602)
*γ*_*i*, 22_	−0.042* (− 1.814)	−0.048** (− 2.098)	−0.044* (− 1.931)
**Variance equation**			
*α*_1_	0.044*** (2.693)	0.077*** (5.202)	0.062*** (4.145)
*α*_2_	0.186*** (6.794)	0.182*** (6.872)	0.183*** (6.829)
*β*_1_	0.949*** (48.420)	0.923*** (67.550)	0.938*** (69.440)
*β*_2_	0.776*** (30.100)	0.778*** (31.160)	0.777*** (30.700)
*θ*_1_	4.783 × 10^− 5^ (0.005)	0.042* (1.928)	2.829 × 10^− 3^ (0.242)
*θ*_2_	0.842 (0.304)	0.499** (2.478)	0.848*** (16.810)

*Notes.* This table shows the estimation results of the DCC-GARCH model. The maximum likelihood estimation is applied, and the estimation method is the two-step approach. The results are converged within 100 iterations. The sample period spans from January 1, 2010, to March 31, 2020. ${R}_{m,t}^c$, ${R}_{a,t}^c$, and ${R}_{d,t}^c$ denote different ${R}_{i,t}^c$ in the mean equation of the DCC-GARCH model. The estimations of the constants in the mean and variance equations are omitted. The *t*-statistics of the elements are shown in parenthesis. One, two and three asterisks (*), respectively, indicate that the *t*-values are significant at the 0.1, 0.05, and 0.01 level. Some less relevant parameter estimates are omitted

Due to the meaning of *γ*_*i*, *mn*_, the off-diagonal elements of the matrix *Γ*_*i*_, the spillover effect from the returns of the SPX to the returns of the CSI300 in the morning and whole day are both positive and significant at the 1% level, while the spillover effect is negative and significant at the 5% level from the return of the SPX to the return of the CSI300 in the afternoon segment. The signs of *γ*_*i*, 12_ indicate that a good performance of the U.S. stock market makes the Chinese stock market perform well the next morning, but poorly the next afternoon. Thus, the impact of the U.S. stock market on the Chinese stock market in the morning and afternoon segments are quite different, which makes the division of morning and afternoon transactions be reasonable and meaningful. In addition, compared with the significance and value of *γ*_*m*, 12_, *γ*_*a*, 12_ is less significant and smaller, which indicates that the impact of the U.S. stock market on the Chinese stock market mainly exists consistently and significantly in the morning transaction.

Apart from testing the significance of return spillovers, the DCC-GARCH can also be applied to measure the strength of the return spillovers. We compute the time-varying conditional correlations ($\frac{h_{i,12t}}{{\left({h}_{i,11t}{h}_{i,22t}\right)}^{1/2}}$), where *h*_11, *t*_ and *h*_22, *t*_ are the conditional variances of the Chinese and U.S. stock markets, and *h*_12, *t*_ is the conditional covariance of both countries. The summary statistics of the conditional correlation estimations are shown in Table 7. The mean of three conditional correlations are all positive and less than 0.2, which indicates that the returns of both markets move in the same direction, but these comovements are not very strong. Both of the means of the conditional correlations, between the SPX and morning trading of CSI300, and between the SPX and whole day trading of CSI300, are almost two times that between the SPX and afternoon trading of CSI300. Therefore, the relationship between the SPX return and the CSI300 return in the morning is greater than that between the SPX and the CSI300 in the afternoon. Comparing these three time-varying conditional correlations’ standard deviations, the conditional correlation between the SPX and morning trading of CSI300 is the most stable. Together with the sign of the minimum conditional correlation, Table 7 implies that the comovements between the SPX and the CSI300 returns in the morning are consistently positive. Again, the linkage between the SPX and the CSI300 over the whole day owes much to the close linkage between the SPX and the CSI300 in the morning trading segment. The linkage between the return of the U.S. market and the Chinese market in the morning transaction daily return and the Chinese morning return is consistent and stable.

**Table 7:** Descriptive statistics of time-varying conditional correlations

	${R}_{m,t}^c$	${R}_{a,t}^c$	${R}_{d,t}^c$
Mean	0.140	0.068	0.138
Std	4.540 × 10^− 7^	0.040	4.911 × 10^− 3^
Min	0.140	−0.017	0.124
Max	0.140	0.146	0.1512

*Notes.* This table reports the summary statistics of the time-varying correlations computed by the DCC-GARCH model. ${R}_{m,t}^c$, ${R}_{a,t}^c$, and ${R}_{d,t}^c$ represent the time-varying conditional correlations between ${R}_t^{s\&p}$ and ${R}_{m,t}^c$, ${R}_{a,t}^c$, and ${R}_{d,t}^c$, respectively

However, the conditional variance of each market in the DCC-GARCH model depends only on its past conditional variance and error. The conditional variance and error of one market cannot impact the conditional variance of the other market. Thus, we require a more general variance function to test the significance of the volatility spillover effects between the two markets.

### Asymmetric BEKK-GARCH model

In the asymmetric BEKK-GARCH model, the mean function is the same as that in the DCC-GARCH model. The variance function is defined by Eq. (8). The conditional variance of each market is determined by the past error, conditional variance, negative error of the two markets, and the past conditional correlation between the two markets.

First, we look at matrix *Γ*_*i*_, which is shown in Table 8, in the mean function to analyze the relationship in terms of the returns across the two markets. There are unidirectional spillover effects between the return of the SPX and the CSI300 in the morning and afternoon segments, respectively. Although the U.S. stock market brings about significant impacts on the Chinese stock market in both the morning and afternoon segments, these two impacts are quite different in terms of the significance, signs, and values. First, as for the significance, the influence of the U.S. stock market on the Chinese stock market in the morning segment is more significant than that on the Chinese stock market in the afternoon segment, which can be verified by the *t*-values of *γ*_*m*, 12_ (14.047) and *γ*_*a*, 12_ (− 2.855). Second, the return spillover from ${R}_t^{s\&p}$ to ${R}_{m,t}^c$ is positive, while the return spillover from ${R}_t^{s\&p}$ to ${R}_{a,t}^c$ is negative, which means tha if the U.S. stock market performs well, then the next-day Chinese stock market is prone to gaining a positive return in the morning transaction but a negative return in the afternoon transaction. Third, the absolute value of *γ*_*m*, 11_ is more than eight times that of *γ*_*a*, 11_, which indicates that the impact of the U.S. stock market on the Chinese stock market exists mainly in the morning transactions. Therefore, it is quite necessary and meaningful for us to divide the daily returns into half-day returns and investigate the return spillovers in the morning and afternoon transactions.

**Table 8:** Asymmetric BEKK-GARCH model estimation results

	${R}_{m,t}^c$	${R}_{a,t}^c$	${R}_{d,t}^c$
**Mean equation**			
*γ*_*i*, 11_	0.013 (0.655)	− 0.161*** (− 8.236)	2.487 × 10^− 3^ (− 0.122)
*γ*_*i*, 12_	0.287*** (14.047)	−0.038*** (− 2.855)	0.247*** (8.962)
*γ*_*i*, 21_	−6.973 × 10^− 3^ (− 0.466)	2.683 × 10^− 3^ (0.167)	−3.091 × 10^− 3^ (− 0.354)
*γ*_*i*, 22_	−0.049** (− 2.110)	−0.045** (− 2.145)	−0.050** (− 2.283)
**Variance equation**			
*a*_*i*, 11_	0.170*** (8.341)	0.266*** (18.900)	0.228*** (12.724)
*a*_*i*, 12_	−0.075** (− 2.735)	− 0.024 (− 1.252)	−0.044*** (− 3.003)
*a*_*i*, 21_	0.025 (1.155)	0.015 (1.440)	−0.050 (− 1.490)
*a*_*i*, 22_	0.053 (1.000)	− 0.025 (− 0.587)	0.019 (0.440)
*b*_*i*, 11_	0.978*** (232.517)	0.962*** (269.475)	0.972*** (271.051)
*b*_*i*, 12_	0.014** (2.240)	3.587 × 10^− 3^ (0.737)	0.012*** (3.094)
*b*_*i*, 21_	−0.029*** (− 4.055)	−0.011** (− 2.072)	−0.041*** (− 4.454)
*b*_*i*, 22_	0.892*** (105.880)	0.897*** (127.187)	0.897*** (120.926)
*d*_*i*, 11_	−0.117*** (− 2.846)	−0.038 (− 0.970)	−0.084** (− 2.369)
*d*_*i*, 12_	−0.080*** (− 3.167)	−0.091*** (− 3.877)	−0.078*** (− 4.879)
*d*_*i*, 21_	−0.077** (− 3.512)	−0.047*** (− 3.134)	−0.127*** (− 4.517)
*d*_*i*, 22_	−0.506*** (− 19.320)	−0.509*** (− 21.449)	−0.489*** (− 19.543)

*Notes.* This table shows the estimation results of the asymmetric BEKK-GARCH model. The maximum likelihood estimation is applied, and the estimation method is Broyden-Fletcher-Goldfarb-Shanno (BFGS) algorithm. The results are converged within 100 iterations. The sample period spans from January 1, 2010, to March 31, 2020. ${R}_{m,t}^c$, ${R}_{a,t}^c$, and ${R}_{d,t}^c$ denote different ${R}_{i,t}^c$ in the mean equation of the asymmetric BEKK-GARCH model. The estimations of the constants in the mean and variance equations are omitted. The *t*-statistics of the coefficients are shown in parenthesis. One, two and three asterisks (*), respectively, indicate that the *t*-values are significant at the 0.1, 0.05, and 0.01 level

Next, we turn to the volatility spillover effects. Table 9 reports the *F*-statistics and illustrates the bidirectional volatility spillover effects between the two markets. Moreover, the volatility spillover from the US to China is due to the U.S. stock market’s spillovers to the Chinese stock market’s morning and afternoon trading segments. Similarly, volatility spillovers from China to the US in both the morning and afternoon contribute to the U.S. stock market’s ability to predict the volatility of the Chinese stock market the next day.

**Table 9:** Test of volatility spillover effects between two countries

	${R}_{m,t}^c$	${R}_{a,t}^c$	${R}_{d,t}^c$
**Spillover direction**			
The US to China (*a*_*i*, 21_ = *b*_*i*, 21_ = *d*_*i*, 21_ = 0)	6.878*** [0.000]	5.105** [0.002]	8.646*** [0.000]
China to the US (*a*_*i*, 12_ = *b*_*i*, 12_ = *d*_*i*, 12_ = 0)	10.945*** [0.000]	5.732*** [0.000]	14.769*** [0.000]

*Notes.* This table reports the *F*-test of the volatility spillover effects based on the estimation results of the asymmetric BEKK-GARCH model. The sample period spans from January 1, 2010, to March 31, 2020. ${R}_{m,t}^c$, ${R}_{a,t}^c$, and ${R}_{d,t}^c$ denote different ${R}_{i,t}^c$ in the mean equation of the asymmetric BEKK-GARCH model. “The US to China” means that the null hypothesis is that there are significant volatility spillover effects from the U.S. to the Chinese stock market. “China to the US” means that the null hypothesis is that there are significant volatility spillover effects from the Chinese to the U.S. stock market. The *p*-value is shown in brackets. One, two and three asterisks (*), respectively, indicate that the *t*-values are significant at the 0.1, 0.05, and 0.01 level

### Robustness checks

To check the robustness of our model, we first divide the sample into four sub-periods: (1) January 1, 2010, to April 15, 2013; (2) April 16, 2013, to June 30, 2018; (3) July 1, 2018, to December 31, 2019; and (4) January 1, 2020, to March 31, 2020.

The model estimations for the first three sub-periods are shown in Tables 10, 11, and 12, respectively. The analysis of the fourth sub-period is introduced in the next section of the case study. Panel A in these four tables captures the return spillovers between these two markets. First, in the first (2010.1.1–2013.4.15) and second (2013.4.16–2018.6.30) sub-periods, there are only positive unidirectional return spillover effects from the U.S. stock market to the Chinese stock market in the morning trading, which means that the return of the U.S. market cannot predict the return of the Chinese market in the afternoon. In the first and second sub-periods, the Chinese market is not able to exert significant influences on the U.S. market. Second, in the third sub-period (2018.7.1–2019.12.31), there are unidirectional return spillovers from the U.S. market to the Chinese market in both the morning and afternoon trading segments. Interestingly, the sign of the return spillover from the U.S. market to the Chinese market in the morning trading is positive, while that to the Chinese market in the afternoon trading is negative, which is consistent with the results during the full sample period (2010.1.1–2019.12.31). Therefore, we can conclude that the U.S. market brought about positive impacts on the Chinese market in the morning trading before June 30, 2018, while after June 30, 2018, the U.S. market can exert positive impacts on the Chinese market in the morning trading and negative impacts on the Chinese market in the afternoon trading.

**Table 10:** Robustness check (January 1, 2010, to April 15, 2013)

	${R}_{m,t}^c$	${R}_{a,t}^c$	${R}_{d,t}^c$
**Panel A: Asymmetric BEKK-GARCH model estimation results**			
**Mean equation**			
*γ*_*i*, 11_	−0.061* (− 1.689)	−0.107*** (− 2.894)	−0.066* (− 1.784)
*γ*_*i*, 12_	0.298*** (9.377)	− 0.026 (− 1.046)	0.280*** (6.224)
*γ*_*i*, 21_	0.028 (− 0.888)	0.060 (1.626)	0.011 (0.476)
*γ*_*i*, 22_	6.856 × 10^− 3^ (− 0.019)	−0.011 (− 0.280)	−0.012 (− 0.292)
**Variance equation**			
*a*_*i*, 12_	−0.054 (− 1.087)	0.056 (1.168)	0.019 (0.432)
*a*_*i*, 21_	0.025 (0.366)	− 0.024 (− 0.909)	5.167 × 10^− 3^ (0.053)
*b*_*i*, 12_	0.098*** (2.950)	0.023 (1.093)	0.075*** (3.614)
*b*_*i*, 21_	−0.035** (− 2.475)	−0.016* (− 1.926)	−0.056*** (− 2.659)
*d*_*i*, 12_	0.041 (0.817)	− 0.025 (− 0.502)	0.035 (0.859)
*d*_*i*, 21_	0.168*** (2.988)	− 0.054** (− 2.097)	0.248*** (3.168)
**Panel B: Test of volatility spillover effects between two countries**			
**Volatility spillovers**			
The US to China (*a*_*i*, 21_ = *b*_*i*, 21_ = *d*_*i*, 21_ = 0)	3.582*** [0.013]	2.268* [0.078]	4.037*** [0.007]
China to the US (*a*_*i*, 12_ = *b*_*i*, 12_ = *d*_*i*, 12_ = 0)	4.742*** [0.003]	0.658 [0.578]	5.730*** [0.000]

*Notes.* Panel A reports the estimation results of the asymmetric BEKK-GARCH model. Panel B reports the *F*-test of the volatility spillover effects based on the estimation results of Panel A

One, two and three asterisks (*), respectively, indicate that the t-values are significant at the 0.1, 0.05, and 0.01 level

**Table 11:** Robustness check (April 16, 2013, to June 30, 2018)

	${R}_{m,t}^c$	${R}_{a,t}^c$	${R}_{d,t}^c$
**Panel A: Asymmetric BEKK-GARCH model estimation results**			
**Mean equation**			
*γ*_*i*, 11_	0.070** (2.197)	− 0.181*** (− 5.731)	0.041 (1.384)
*γ*_*i*, 12_	0.222*** (5.985)	3.531 × 10^− 4^ (− 0.014)	0.251*** (5.701)
*γ*_*i*, 21_	5.226 × 10^− 3^ (0.263)	− 0.035 (− 1.606)	−0.011 (− 0.752)
*γ*_*i*, 22_	−0.079** (− 2.505)	−0.077*** (− 2.601)	−0.081** (− 2.405)
**Variance equation**			
*a*_*i*, 12_	−0.095*** (− 3.528)	−0.033 (− 1.231)	−0.059*** (− 3.435)
*a*_*i*, 21_	0.132*** (4.207)	5.452 × 10^− 4^ (0.025)	− 0.105*** (− 2.620)
*b*_*i*, 12_	6.751 × 10^− 3^ (0.773)	5.076 × 10^− 3^ (0.713)	0.014*** (2.663)
*b*_*i*, 21_	−0.040* (− 1.876)	0.015 (1.151)	− 9.131 × 10^− 3^ (− 0.412)
*d*_*i*, 12_	−0.099*** (− 3.183)	0.101*** (3.361)	− 0.062*** (− 2.783)
*d*_*i*, 21_	−2.427 × 10^− 4^ (5.140 × 10^− 3^)	−0.011 (− 0.332)	−0.022 (− 0.385)
**Panel B: Test of volatility spillover effects between two countries**			
**Volatility spillovers**			
The US to China (*a*_*i*, 21_ = *b*_*i*, 21_ = *d*_*i*, 21_ = 0)	6.632*** [0.000]	0.724 [0.537]	2.487* [0.059]
China to the US (*a*_*i*, 12_ = *b*_*i*, 12_ = *d*_*i*, 12_ = 0)	8.730** [0.000]	5.199*** [0.001]	8.827*** [0.000]

*Notes.* Panel A reports the estimation results of the asymmetric BEKK-GARCH model. Panel B reports the *F*-test of the volatility spillover effects based on the estimation results of Panel A

One, two and three asterisks (*), respectively, indicate that the t-values are significant at the 0.1, 0.05, and 0.01 level

**Table 12:** Robustness check (July 1, 2018, to December 31, 2019)

	${R}_{m,t}^c$	${R}_{a,t}^c$	${R}_{d,t}^c$
**Panel A: Asymmetric BEKK-GARCH model estimation results**			
**Mean equation**			
*γ*_*i*, 11_	−0.028 (− 0.552)	−0.194*** (− 4.011)	−0.046 (− 0.934)
*γ*_*i*, 12_	0.359*** (5.928)	− 0.100*** (− 3.323)	0.317*** (4.275)
*γ*_*i*, 21_	5.627 × 10^− 3^ (− 0.166)	−0.012 (− 0.201)	8.246 × 10^− 3^ (0.280)
*γ*_*i*, 22_	−0.075 (− 1.250)	−0.045 (− 0.765)	−0.063 (− 1.014)
**Variance equation**			
*a*_*i*, 12_	0.140** (2.382)	0.056 (0.975)	−0.042 (− 1.168)
*a*_*i*, 21_	0.058 (0.332)	− 0.015 (− 0.483)	−0.100 (− 1.485)
*b*_*i*, 12_	−0.169** (− 2.329)	0.046 (0.328)	0.024*** (3.080)
*b*_*i*, 21_	0.234 (1.569)	− 0.045 (− 0.529)	−0.044** (− 1.985)
*d*_*i*, 12_	0.119* (1.931)	0.415*** (5.666)	0.191*** (6.026)
*d*_*i*, 21_	0.273** (2.290)	0.046 (1.303)	−0.174** (− 2.156)
**Panel B: Test of volatility spillover effects between two countries**			
**Volatility spillovers**			
The US to China (*a*_*i*, 21_ = *b*_*i*, 21_ = *d*_*i*, 21_ = 0)	3.540** [0.014]	0.760* [0.516]	2.784** [0.039]
China to the US (*a*_*i*, 12_ = *b*_*i*, 12_ = *d*_*i*, 12_ = 0)	16.011*** [0.000]	11.737*** [0.000]	19.234*** [0.000]

*Notes.* Panel A reports the estimation results of the asymmetric BEKK-GARCH model. Panel B reports the *F*-test of the volatility spillover effects based on the estimation results of Panel A

One, two and three asterisks (*), respectively, indicate that the t-values are significant at the 0.1, 0.05, and 0.01 level

However, the variance functions are not robust. The significances of the *F-*statistics of the volatility spillovers between the US and China in the afternoon trading in Tables 10, 11 and 12 are not consistent. Therefore, the volatility spillover effects between the two markets in this regard require further research.

### A case study of COVID-19

On December 31, 2019, a pneumonia of unknown origin was detected in Wuhan (according to the World Health Organization (WHO) website^2^). On January 23, 2020, Wuhan was put on lockdown, and China shifted to an anti-pandemic period shortly afterward. On January 30, 2020, the outbreak was declared a Public Health Emergency of International Concern by the WHO. In 1 week, from January 23 to February 2, the CSI300 index decreased by 7.88%. On February 27, a person was first detected carrying COVID-19 in the US, and between February 27 and March 23, the SPX decreased by over 28%.

Because the COVID-19 pandemic occurred so suddenly and so recently, how it has impacted the stock markets is not fully covered in previous empirical studies. By conducting the case study, we aim to fill this gap to some extent by providing the most recent empirical evidence of the impact of COVID-19 on the capital markets, especially on the interactions between the stock markets across the countries. Thus, we conduct a case study of the impact of the COVID-19 pandemic using the daily stock transactions from January 1, 2020, to March 31, 2020. Please refer to the results in Table 13. First, we find results that consistent with the fore-stated main conclusions of the paper. In other words, during this period, the U.S. market still has positive spillover to China’s next day’s morning trading. Second, and most importantly, the Chinese stock market exerted significant return spillovers back onto the U.S. stock market during that period, which reflects the transmission of the serious negative impact of COVID-19 between markets.

**Table 13:** Robustness check (January 1, 2020, to March 31, 2020)

	${R}_{m,t}^c$	${R}_{a,t}^c$	${R}_{d,t}^c$
**Panel A: Asymmetric BEKK-GARCH model estimation results**			
**Mean equation**			
*γ*_*i*, 11_	−0.030 (− 0.323)	4.765 × 10^− 6^ (− 2.856 × 10^− 5^)	− 0.242* (− 1.811)
*γ*_*i*, 12_	0.219*** (5.241)	− 0.080*** (− 2.893)	0.145** (2.351)
*γ*_*i*, 21_	−0.064 (− 0.740)	−0.110 (− 0.995)	−0.136*** (− 2.634)
*γ*_*i*, 22_	−0.303*** (− 2.763)	−0.461*** (− 7.004)	−0.399*** (− 8.016)
**Variance equation**			
*a*_*i*, 12_	−0.153 (− 1.323)	0.294 (1.015)	2.786 × 10^− 3^ (0.041)
*a*_*i*, 21_	−0.120 (− 1.588)	0.130** (2.015)	0.157 (0.838)
*b*_*i*, 12_	0.022 (0.497)	0.513*** (3.819)	−0.178 (− 1.607)
*b*_*i*, 21_	2.823 × 10^− 3^ (0.065)	0.048** (2.006)	0.155*** (2.811)
*d*_*i*, 12_	0.227* (1.887)	− 0.146 (− 0.343)	−0.040 (− 0.619)
*d*_*i*, 21_	−0.143 (− 1.145)	−0.040 (− 0.325)	5.360 × 10^− 3^ (− 0.023)
**Panel B: Test of volatility spillover effects between two countries**			
**Volatility spillovers**			
The US to China (*a*_*i*, 21_ = *b*_*i*, 21_ = *d*_*i*, 21_ = 0)	2.749** [0.041]	3.950** [0.008]	3.866*** [0.009]
China to the US (*a*_*i*, 12_ = *b*_*i*, 12_ = *d*_*i*, 12_ = 0)	2.407* [0.065]	5.454*** [0.001]	0.986 [0.398]

*Notes.* Panel A reports the estimation results of the asymmetric BEKK-GARCH model. Panel B reports the *F*-test of the volatility spillover effects based on the estimation results of Panel A

One, two and three asterisks (*), respectively, indicate that the t-values are significant at the 0.1, 0.05, and 0.01 level

We provide the most recent empirical evidence of the impact of COVID-19 on the capital markets and the interactions between stock markets across countries. Moreover, this case study has potentially important implications for policy-makers to coordinate global integration regulatory policies. As the spillover effects are strengthened between the U.S. and Chinese stock markets, crisis and volatility will also become more contagious between different markets. The case study of COVID-19 in this paper provides direct evidence of the spillover effects of turmoil or crisis from one country to another. In the context of macro-prudential regulation, our results highlight the importance of strengthening the coordination of the policies in different countries and maintaining financial stability internationally.

### Explanations for increasingly stronger spillovers

In the previous sections, we provide evidence that the return spillovers between the two markets are becoming stronger and stronger in recent years. It is necessary for us to investigate the potential reasons behind this stronger spillover phenomenon. Foreign access to Chinese capital market has always remained under tight control with important innovation in recent years. On November 17, 2014, Shanghai-Hong Kong Stock Connect was launched to provide a cross-boundary investment channel that connects the Shanghai Stock Exchange and the Hong Kong Stock Exchange. Two years later, Shenzhen-Hong Kong Stock Connect was launched on December 5, 2016, broadening the range of A-shares that international investors can trade. These two channels have an enormous impact in that Hong Kong and international capital can more easily invest in stocks in the Chinese stock market, and Chinese domestic capital can also exert influences onto international stock markets, including the Hong Kong, New York, and London markets, et cetera. This change significantly enhances the nexus between the Chinese and U.S. stock markets through capital flows and trading volume. We examine the impacts of the capital flows of Shanghai-Hong Kong Stock Connect and Shenzhen-Hong Kong Stock Connect on the spillover intensity between the two markets. Although investors in both domestic and foreign jurisdictions will benefit, the northbound channel for foreign investment into Shanghai’s equity markets is seen as the more significant development, given the global implications of China’s financial reform trajectory and the tight regulations that governed such investment into China previously.

**Fig. 4:**
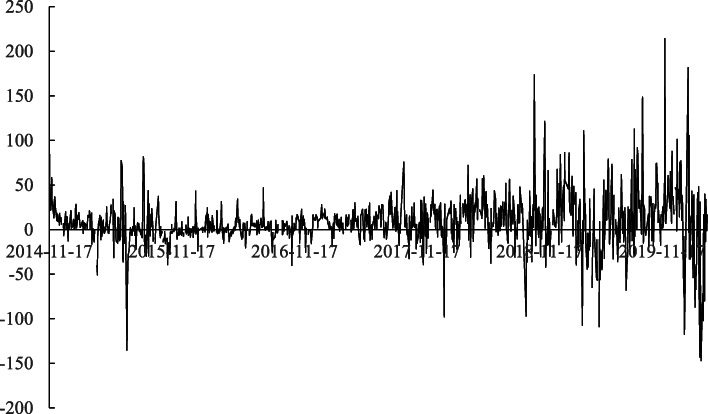
Daily net cash flow of the Northbound Capital. *Notes*. This figure plots the daily net cash flow of the Northbound Capital. The data are from the Wind database and span from November 17, 2014, to March 31, 2020

We download the daily net cash flow of the Northbound Capital volume from the Wind database. From Fig. 4, it can be seen that the daily capital flow volume is increasing over time as the daily limit is eased by the Chinese government step by step.^3^ Moreover, the volatility of the daily net cash flow of the Northbound Capital is increasing as time goes by, which reflects the Northbound Capital becoming increasingly active in trading and shifting stock holdings in the Chinese A-share stock market.

By following Lee and Tong (2018) and Jiang et al. (2018), we construct three empirical tests to investigate how Stock Connects as well as Northbound Capital flow influence the spillover effects:

9 $$ {Corr}_t={\beta}_0+{\beta}_1{Launch}_t+{\varepsilon}_t, $$

10 $$ {Corr}_t={\beta}_0+{\beta}_1{Flow}_t+{\varepsilon}_t, $$

11 $$ {Corr}_t={\beta}_0+{\beta}_1{Vol}_t+{\varepsilon}_t, $$

where *Corr*_*t*_ denotes the time-varying conditional correlation between the daily returns of the CSI300 and SPX, which is calculated in the DCC-model as is shown in Table 7. *Launch*_*t*_ is a dummy variable, and it is 1 when *t* is after November 17, 2014, the time when Shanghai-Hong Kong Stock Connect was first launched; otherwise, it is 0. *Flow*_*t*_ denotes the absolute value of the daily net cash flow of the Northbound Capital, whose unit is 10 billion RMB. *Vol*_*t*_ represents the monthly sample variance of the daily net flow of the Northbound Capital.

The results in Table 14 show that conditional correlations between the Chinese and U.S. stock markets after the launch of the Shanghai-Hong Kong Stock Connect are significantly higher than those before the launch of the Shanghai-Hong Kong Stock Connect. Thus, Stock Connects do exert a positive impact on the increasingly stronger spillovers between the two markets. Moreover, the second row of Table 14 shows that the absolute value of the daily net cash flow of the Northbound Capital can bring about significant positive influences on the correlation between the two markets, which measures the intensity of the return spillovers between the two markets. The increasing absolute value of the daily net cash flow of the Northbound Capital contributes to the stronger return spillovers between the two markets. According to Fig. 4, the absolute value of the daily net cash flow of the Northbound Capital becomes higher over time. Therefore, there are stronger return spillovers. Furthermore, the coefficient on the third row of Table 14 shows that *Vol*_*t*_ exerts a significant positive influence on the correlation between the two markets, also. There are stronger daily return spillovers between the two markets when the monthly volatilities of the daily net flow of Northbound Capital are higher. Combined with the trend in Fig. 4, we find that the volatility of the daily net flow of Northbound Capital is indeed increasing over time, which indicates that the conditional correlations between the two markets are increasingly high. Therefore, the return spillovers become stronger and stronger. Based on these empirical results, we provide one possible explanation in that the launches of Stock Connects, the higher daily net cash flow of the Northbound Capital, and the higher volatility of the Northbound Capital cash flow all contribute to the stronger return spillovers between these two markets. There could exist other factors that play a role in the increasingly stronger spillovers at the same time, which is a topic that is worthy of further exploration.

**Table 14:** Stock connects and spillover effects

	Time-varying conditional correlation between the daily returns of CSI300 and SPX (*Corr*)		
*Launch*	1.097 × 10^− 3^*** (1.993 × 10^− 4^)		
Northbound Capital volume (*Flow*)		1.341 × 10^− 4^** (6.550 × 10^− 5^)	
Northbound Capital volatility (*Vol*)			9.950 × 10^−5^***s (1.640 × 10^− 5^)
_*R*_^2^	0.012	0.003	0.029
Observations	2406	1241	1241

*Notes.* This table reports three regressions corresponding to Eqs. (9), (10) and (11). Row 1 (Eq. (9)) represents the regression of the time-varying conditional correlation between the daily returns of CSI300 and SPX on a dummy variable *Launch*. The dummy variable *Launch* is 1 when *t* is after November 17, 2014, the time when Shanghai-Hong Kong Stock Connect was first launched; otherwise, it is 0. Row 2 (Eq. (10)) stands for the regression of the time-varying conditional correlation between the daily returns of CSI300 and SPX on the absolute value of the daily net flow of the Northbound Capital (*Flow*), which is denoted in RMB10 billion. Row 3 (Eq. (11)) stands for the regression of the time-varying conditional correlation between the daily returns of CSI300 and SPX on the monthly sample variance of the daily net flow of the Northbound Capital (*Vol*). We download the required data from the Wind database. The sample period of Eq. (9) spans from November 17, 2014, to March 31, 2020. The sample period of Eqs. (10) and (11) spans from January 1, 2010, to March 31, 2020. Standard errors are reported in parentheses. One, two, and three asterisks (*) indicate the *t*-values are significant at the 0.1, 0.05, and 0.01 level, respectively

## Conclusions

This paper investigates the return and volatility spillover effects between the Chinese and U.S. stock markets during the period from 2010 to 2019, and January to March, 2020 (COVID-19 pandemic period), respectively. A DCC-GARCH model is used to test the return spillover effects and describe the comovements between these two markets. Further, an asymmetric BEKK-GARCH model is applied to examine both the return and volatility spillover effects.

The empirical results indicate that there are only positive significant unidirectional return spillover effects from the U.S. stock market to the Chinese stock market during 2010 to 2020. In contrast to previous studies on the daily spillover effects between these two markets, we investigate the intraday transmission mechanism. To be specific, the return spillovers from the US to China exist only in the morning transaction but not in the afternoon during the period of January 1, 2010 to June 30, 2018, which means that investors complete their responses to information from the U.S. stock market in semi-day trading. However, both the Chinese stock market’s morning and afternoon trading begin to be affected by the U.S. stock market after June 30, 2018. These two return spillovers are quite different in terms of their significance, signs, and values. More specifically, by comparing the *t*-values, signs and absolute values of these two types of return spillovers, we can conclude that the impact of the U.S. stock market on the Chinese stock market mainly exists in the morning transaction of the Chinese market during the period June 30, 2018, to December 31, 2019. Therefore, it is necessary and meaningful for us to divide daily returns into half-day returns and investigate the spillovers in the Chinese stock market’s morning and afternoon trading segments. For the COVID-19 pandemic period (January to March, 2020), apart from the return spillovers from the U.S. to China’s morning and afternoon trading, there is also a significant impact in travelling from the Chinese stock market to the U.S. stock market, which indicates bidirectional return spillovers between these two markets during January to March, 2020. One possible explanation is that COVID-19 was first discovered and broke out in China, and thus, the Chinese stock market was significantly affected by the pandemic, and the U.S. stock market was influenced later.

Evidence from the asymmetric BEKK-GARCH model shows that there are significant bidirectional volatility spillover effects between these two markets. The volatility spillover effects exist in both the morning and afternoon transactions. However, the results of the volatility spillover effects are not robust in different samples. Why this inconsistency exists is an interesting question for further investigation.

## Figures and Tables

**Fig. 5:**
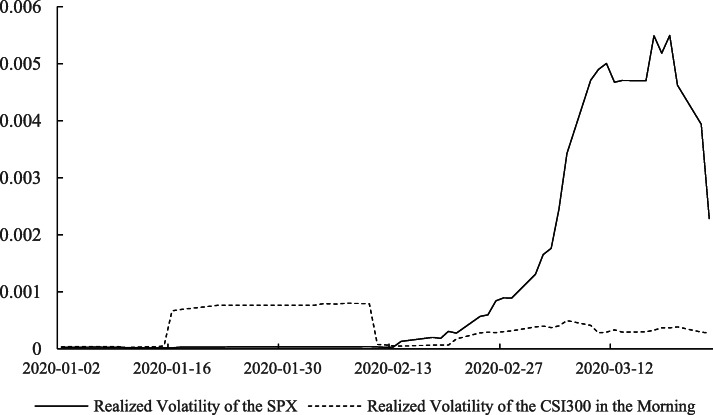
Realized volatility of the SPX and CSI300 in the morning. *Notes.* This figure plots the realized volatilities of CSI300 in the morning and SPX over the whole day. The data are from the Wind database and span from January 1, 2020 to March 31, 2020

**Fig. 6:**
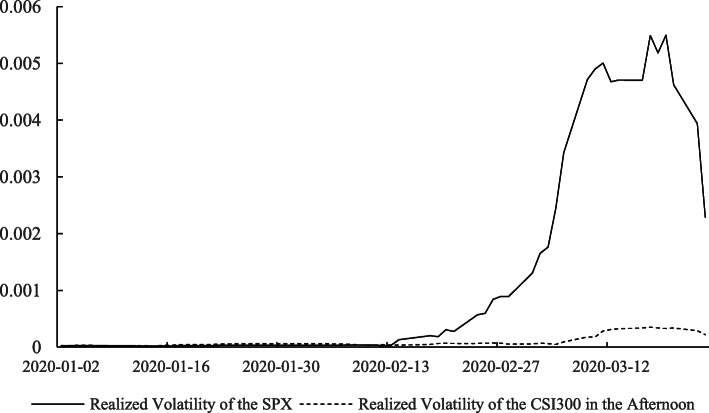
Realized volatility of the SPX and CSI300 in the afternoon. *Notes.* This figure plots the realized volatilities of CSI300 in the afternoon and SPX over the whole day. The data are from the Wind database and span from January 1, 2020 to March 31, 2020

**Fig. 7:**
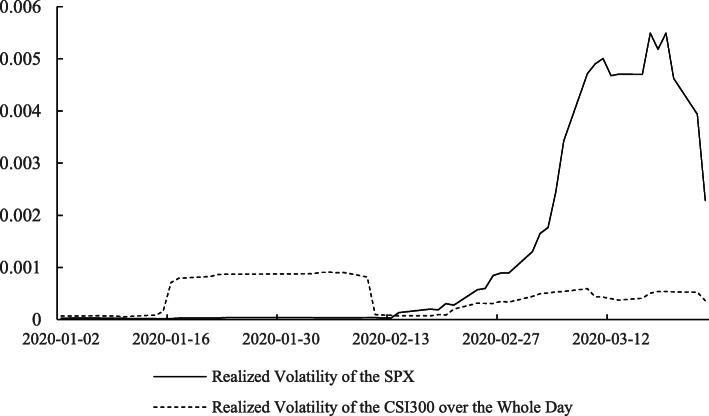
Realized volatility of the SPX and CSI300 over the whole day. *Notes.* This figure plots the realized volatilities of CSI300 and SPX over the whole day. The data are from the Wind database and span from January 1, 2020 to March 31, 2020

## Data Availability

The authors declare that all the supporting data of this study is from public sources, which are available and feasible for everyone. The data about closing price of the indices of the Chinese and U.S. stock markets is available in Wind and Bloomberg database, respectively.

## Appendix

**Table 15:** Asymmetric BEKK-GARCH model estimation result

	$$ {R}_{d,t}^c $$
**Mean equation**	
*γ*_*i*, 11_	−0.038 (− 1.463)
*γ*_*i*, 12_	−0.062** (− 2.077)
*γ*_*i*, 21_	4.130 × 10^− 4^ (0.052)
*γ*_*i*, 22_	0.060*** (2.622)
**Variance equation**	
*a*_*i*, 12_	2.877 × 10^− 3^ (0.481)
*a*_*i*, 21_	−0.014 (− 0.589)
*b*_*i*, 12_	6.793 × 10^− 3^ (− 0.494)
*b*_*i*, 21_	1.405 × 10^− 3^ (− 0.284)
*d*_*i*, 12_	0.025*** (2.844)
*d*_*i*, 21_	0.050 (1.571)
**Volatility spillovers**	
The US to China (*a*_*i*, 21_ = *b*_*i*, 21_ = *d*_*i*, 21_ = 0)	1.355 [0.254]
China to the US (*a*_*i*, 12_ = *b*_*i*, 12_ = *d*_*i*, 12_ = 0)	3.810*** [0.009]

*Notes.* This table shows the robustness check of the first sample sub-period (from November 25, 1994, to September 28, 2001), which is the sample period in the Wang and Firth (2004) paper

The intraday trading data are not available until 2007. Thus, when we examine the period from 1994.11.25 to 2001.9.28 as in Wang and Firth (2004), we look only at the all-day trading spillover effects. We are not able to distinguish between the morning trading and afternoon trading

One, two and three asterisks (*), respectively, indicate that the t-values are significant at the 0.1, 0.05, and 0.01 level

## Footnotes

1 The U.S. stock market’s spillover is inconsistent on Chinese stock market’s next day’s afternoon trading during different time periods. To be more specific, during the period of 2010 to mid-2018, there is no spillover from the U.S. market to the Chinese market’s next day’s afternoon trading. However, during the period of mid-2018 to 2020, the return spillover from the U.S. stock market to the Chinese stock market’s afternoon trading became negative. Why there is inconsistency during different periods requires further research.

2 https://www.who.int/emergencies/diseases/novel-coronavirus-2019/events-as-they-happen

3 http://www.sse.com.cn/services/hkexsc/disclo/announ/c/c_20180411_4498499.shtml
